# From the Front Lines of the Appalachian Addiction Crisis: Healthcare Providers Discuss Opioids, Meth and Recovery

**DOI:** 10.13023/jah.0204.09

**Published:** 2020-09-01

**Authors:** Carl G. Leukefeld

**Affiliations:** University of Kentucky

**Keywords:** Appalachia, book review, addiction, opioids, drug use, recovery

## Abstract

The *Journal of Appalachian Health* is dedicated to reviewing various types of media related to contemporary concepts that affect the health of Appalachia. As the opioid-related overdose deaths ravish Appalachia, now more than ever, we each must devote energy to understanding addiction and pathways to recovery. Dr. Carl Leukefeld reviews the book *From the Front Lines of the Appalachian Addiction Crisis: Healthcare Providers Discuss Opioids, Meth and Recovery*.

## MEDIA TYPE: BOOK

### CITATION

Welch W. *From the Front Lines of the Appalachian Addiction Crisis Healthcare Providers Discuss Opioids, Meth and Recovery*. Jefferson, North Carlina: McFarland & Company, Inc. 2020.

ISBN-13: 978-1476682266; Cost: $39.95 paperback, $14.39 Kindle edition

### ABOUT THE REVIEWER

Carl Leukefeld is Professor and past Chair of the University of Kentucky Department of Behavioral Science. He is the founding Director of the Center on Drug and Alcohol Research who came to the University of Kentucky to establish the Center from the National Institute on Drug Abuse. He was also the Chief Health Services Officer of the United States Public Health Service. Dr. Leukefeld has over 350 publications.

### ABOUT THE AUTHOR

This book is a compilation of stories about the harsh realities of opioid addiction in Appalachia which include poverty, health disparities, family issues, limited work income, interpersonal violence, and few close places for help. The stories are told by those who care—health providers, professors, teachers, interventionists, physicians, and concerned community members.

### THE REVIEW

If you want to learn about the opioid crisis in Appalachia, pick up this book. From self-help groups like Alcoholics Anonymous (AA), to Medications Assisted Treatment, to faith-based interventions, and to community efforts, this collection of stories lets readers better understand Appalachia’s current addictions crisis. Stories and storytelling are part of Appalachia’s traditions and history of the grit and determination. Early stories include making moonshine and being pursued by revenuers. Songs, ballads, and story-telling seminars reinforce that tradition today in Appalachia. Growing marijuana made parts of Appalachia a target in the federal drug supply story as did shaking and baking to make meth. This has changed. Opioid use is not like hillbilly moonshining or growing illegal marijuana. As the stories in this book unfold, the deadly realities are brought to life by those who care and have taken actions against opioids in spite of limited local community treatment and prevention resources. These are not big city stories like those about heroin, cocaine, and speed balling several decades ago. These stories are told by those who care—health providers, professors, teachers, interventionists, physicians, and concerned community members including writers. Most of these stores are common for many Appalachians. They embrace opioid addiction through the lenses of biology and the physiology of addiction, highlighting the complications of Medications Assisted Therapy (MAT) like naltrexone; the psychological insights of care providers; the social/environmental planning of cities like Huntington, West Virginia to meet opioid addiction head on; and a spiritual framework used in faith based communities. Addiction is also examined as a brain disease with distinct dependency characteristics including physical, psychological and tolerance. Jails and prisons are also mentioned.

Like most of my East Kentucky friends who live near our farm, we know someone who has been touched by opioids. The life struggles of opioid users in East Kentucky are like those in the southern region of Appalachia, including West Virginia and Southwest Virginia. These stories present the harsh reality of addiction associated with poverty, health disparities, family issues, limited work income, interpersonal violence, and few close places for help. Questions that continue to nag me as I read these stories are: How can we be respectfully motivate and engage users to be hopeful? How can we encourage folks to engage in the recovering process rather than a cycle of relapse? How can we help others understand that opioid addiction is a chronic and relapsing disorder? Using stories as an intervention is an approach our group developed, with NIDA support, that we called Structured Stories. This cognitive behavioral harm reduction approach uses stories developed by East Kentucky users and caregivers in focus groups. Each story presents a real-life situation that puts a user in a high-risk place. The stories are not complete but are completed by the participant. The response behavior is then examined by looking at what came before it (the person’s thoughts) and what came after (the consequences). Then alternative thoughts and behaviors are explored. My hope is that readers are inspired by the stories in this book to creatively support and help like the storytellers in this book.

Publisher link:https://mcfarlandbooks.com/product/from-the-front-lines-of-the-appalachian-addiction-crisis/

